# Sensitive Time-Resolved Fluorescence Immunoassay for Quantitative Determination of Oxyfluorfen in Food and Environmental Samples

**DOI:** 10.3389/fchem.2020.621925

**Published:** 2021-01-07

**Authors:** En Ze Sheng, Yu Ting Tan, Yu Xiao Lu, Yue Xiao, Zhen Xi Li

**Affiliations:** ^1^Jiangsu Collaborative Innovation Center of Biomedical Functional Materials and Jiangsu Key Laboratory of Biofunctional Materials, School of Chemistry and Materials Science, Nanjing Normal University, Nanjing, China; ^2^Department of Pesticide Science, College of Plant Protection, Nanjing Agricultural University, Nanjing, China

**Keywords:** oxyfluorfen, time-resolved fluorescence immunoassay, europium, pesticide residue, high throughput detection

## Abstract

The direct and indirect competition time-resolved fluorescence immunoassays (dc-TRFIA, ic-TRFIA) were established by combining the autofluorescence properties of lanthanide europium (Eu) with the monoclonal antibody of oxyfluorfen. The purified Eu antibody was optimized and the conditions such as the working concentration of the Eu antibody, monoclonal antibody, and working buffer were optimized. In the optimal condition, the IC_50_ of dc-TRFIA was 10.27 ng/mL, the lowest detection limit IC_10_ was 0.071 ng/mL, the detection range (IC_10_-IC_90_) was 0.071–1074.3 ng/mL, and the detection range (IC_10_-IC_90_) and IC_50_ of ic-TRFIA were 0.024–504.6 and 2.76 ng/mL, respectively. The comparison showed that the sensitivity and detection limit of ic-TRFIA were superior to dc-TRFIA. The cross reaction (CR) tests showed that the CR with other oxyfluorfen structure analogs was <0.02%, except that there was a certain CR with the benzofluorfen (CR = 11.58) and the bifenox (CR = 8.23%). The average recoveries of ic-TRFIA were 74.6–108.3%, and the RSDs were between 2.1 and 10.9%, in the addition recovery test with five substrates. The results of the correlation test with the real samples of GC-ECD showed that they were highly correlated (*y* = 0.975x – 0.4446, *R*^2^ = 0.9901), which proved that the TRFIA method established in this study had high reliability and accuracy and could be used in environment and agricultural products for rapid detection of oxyfluorfen residues.

## Introduction

Oxyfluorfen [2-chloro-α, α, α-trifluoro-p-tolyl-(3-ethoxy-4-nitro-phenyl)] is a kind of fluorinated diphenyl ether herbicide that developed by the Rohm and Hass (Sheng et al., 2020). It is mainly used to inhibit weed photosynthesis for achieving herbicidal purpose (Wang et al., 2016). The drug is used in a wide-range, broad weed control spectrum, long duration, high activity, and a variety of combination with other herbicides. There is no residual toxicity to the succeeding crops, and it has a low toxicity to humans and animals, but it's highly toxic to aquatic invertebrates, wildlife, and fishes (Hirakawa et al., 2015; Catalá-Icardo et al., 2017; dos Santos et al., 2019). What is more, oxyfluorfen cannot be metabolized by plants; it can only be slowly assimilated by microorganisms, and this issue has gradually attracted people's attention. Such as the United States Food and Drug Administration (FDA) and Health Canada have revised the maximum residue limit (MRL) of oxyfluorfen twice in 3 years (2014 and 2017) (Sheng et al., 2018). So, it is necessary to establish rapid, highly sensitive, economical, and friendly methods for the detection of residual oxyfluorfen. At present, the detection method of oxyfluorfen is limited to the method of instrumental analysis [such as high-performance liquid chromatography (HPLC), gas chromatography, HPLC-MS, etc.] (Xiang et al., 2002; Wang et al., 2007, 2012; Calderon et al., 2015); however, there are some problems by instrument detection, such as high consumption (the instrument is very expensive), high pollution (dosage of organic solvent, not environmentally friendly), complex operation of the program, and difficulties of realizing high-throughput detection (Bettencourt Da Silva et al., 2012; López-Blanco et al., 2018; Yang et al., 2018; Drabova et al., 2019). The immune analysis is a simple, rapid, sensitive, low-cost, high-throughput detection technology that has become one of the hotspots and development trends in the field of food and environmental products quality safety testing (Watanabe et al., 2004; Brian et al., 2009; Navarro et al., 2013). The time-resolved fluorescence immunoassay (TRFIA) method uses the lanthanide elements with unique fluorescence characteristics and the corresponding chelates as markers to label the target antibody or antigen (Zhang et al., 2013; Li et al., 2014). After the immune response is over, the enhancement solution is added to dissociate-chelation, the formation of a new chelate that can emit higher fluorescence intensity (the fluorescence intensity is proportional to the amount of the labeled substance), use a TRFIA detector to delay the detection time, and eliminate the interference of background fluorescence. Fluorescence intensity is realizing quantitative detection of analytes (Gui et al., 2009; Huang et al., 2012; Xu et al., 2012; Zhang et al., 2013; Sheng et al., 2016). The unique fluorescence characteristics of lanthanides make TRFIA a new immunoassay method with high sensitivity, no interference from background fluorescence, broad spectrum, large Stokes shift, dissociation enhancement, and no radioactivity (Shi et al., 2015). TRFIA, as a new label detection technology that only emerged in the 1990s, has been widely used in medical, environmental, and agricultural pollutant detection methods, and it is bound to bring new vitality to label immunoassay technology.

In this paper, the europium ion (Eu^3+^) in the lanthanide element was selected as a marker, and the synthetic oxyfluorfen monoclonal and goat anti-mouse secondary antibodies were respectively labeled to establish the dc- of oxyfluorfen. The TRFIA and ic-TRFIA methods are applied to the actual detection of food and environmental samples, and the established TRFIA is analyzed by gas chromatography-electron capture detection (GC-ECD). The ultra-sensitive TRFIA analysis method established in this study will provide new research ideas and technical means for the ultra-sensitive detection of pesticide residues and other small molecule residues, and will provide necessary for the subsequent development of a TRFIA kit with independent property rights.

## Materials and Methods

### Reagents

Oxyfluorfen and its structural analogs were purchased from Jiangsu Suke Agrochemical Co., Ltd. The N_0_-[p-isothiocyanato-benzyl]-diethylene-triamine-N_1_, N_2_, N_3_, N_4_-tetraacetate-Eu^3+^ was purchased from Tianjin institute of radiation medicine. Goat anti-mouse IgG antibody was purchased from Sigma company. The oxyfluorfen monoclonal antibody were laboratory homemade. The Sepharose CL-6B agarose gel was obtained from Beijing Qisong biological technology Co., Ltd. The PD-10 evolutionary column was purchased from American PE company. The fluorescent enhancement fluid was obtained from Jiangsu Institute of Atomic Medicine. All the other regents were analytically pure reagents.

### Instruments

The TRFIA value were measured by Spectra Max M5 (Molecular Devices, Sunnyvale, CA, USA). An Agilent 7890A GC equipped with an ultraviolet detector (Agilent, Wilmington, DE, USA) was used to verify the accuracy of the TRFIA. Milli-Q purified water was obtained from a Milli-Q purification system (Millipore, Bedford, MA, USA). A 96-well polystyrene fluorescent plate (Jiangsu Institute of Atomic Medicine) was used as a reaction vessel for the TRFIA.

### Preparation for Eu Markers

The preparation method of the Eu marker was according to the previous articles (Sheng et al., 2016). Briefly, 10 mg of the oxyfluorfen monoclonal antibody was dissolved in 1 mL phosphate buffer saline (PBS) (50 mmol/L, pH 7.5) solution, and the buffer conditions were changed by PD-10 column. Then 1.0 mL of the antibody solution was taken from the converted buffer, mixed with 0.5 mg Eu^3+^-DTTA, and then the solution was stirred by magnetic agitation in darkness for 24 h at 4°C. The reaction solution was purified by a Sepharose CL-6B column (1 × 40 cm) using an 80 mmol/L Tris-HCl buffer of pH 7.8 as an eluent, and the eluate was received at a 200 μL/tube. The fluorescence value and UV absorption value of 280 nm were monitored. The solution with higher fluorescence and UV absorption was collected as the purified Eu standard primary antibody. The glycerol and 0.2% BSA was added as an equal volume, then the solutions were stored at −20°C.

Goat anti-mouse IgG (secondary antibody) and Eu^3+^-DTTA coupling method was similar to the above method. After 1 mL of goat anti-mouse IgG (10 mg/mL) was converted to buffer conditions on a PD-10 column, it was eluted with carbonate-buffered saline (CBS) buffer and diluted to a concentration of 2 mg/mL, and 500 μL of the converted IgG solution was added to 0.2 mg Eu^3+^-DTTA, and the mixture was stirred for 20 h at 25°C in the dark. Purified by Sepharose CL-6B column (1 × 40 cm), a 200 μL/tube received the eluent. The fluorescence values and uv absorption values at 280 nm were monitored. The solution with both high fluorescence intensity and UV absorption value was collected as a purified EU-labeled secondary antibody, added an equal volume of glycerol and 0.2% BSA to the collected solution, and then stored at −20°C.

### Procedure of TRFIAs

For dc-TRFIA, microplates were coated with coating antigen (100 μL/well, in CBS) overnight at 4°C. The plates were washed five times with Tris-HCl buffer containing 0.05% Tween-20 (TBST) and blocked by incubating with 1% ovalbumin (OVA) in Tris-HCl buffer (TBS) (250 μL/well) for 0.5 h at 37°C. After another washing step, either the samples or standard in TBS was added followed by addition of the diluted Eu^3+^-labeled McAb (50 μL/well) together for 1 h at 37°C. After the wash step, an enhancement solution (250 μL/well) was added into the plates. Then the SpectraMax M5 was used to detect the fluorescence intensity (F) after 10 min of the mechanical shaking.

For the ic-TRFIA, after the coating and blocking steps formed as above, a volume of 50 μL/well standard or sample solution in TBS together with 50 μL of McAb in TBS was added into the plate. The plate was incubated for 1 h at 37°C. After the further wash step, the diluted Eu^3+^-labeled Goat-IgG (100 μL/well) was dispensed into each well and incubated for 1 h at 37°C. After washing the plate again, the enhancement solution was added, and the F was measured.

Each of the samples was tested in triplicate, and the mean value of F/F_0_–the ratio of the fluorescence signal with oxyfluorfen (F) to that without oxyfluorfen (F_0_)—was plotted on competitive curves. The half-maximal inhibition concentration (IC_50_) and the limit of detection (LOD) were obtained from a four-parameter logistic equation of the sigmoidal cures using the Origin Pro 8.0 software.

### Optimization

The factors that had a great influence on the sensitivity of the TRFIA analysis method are optimized: the original concentration of the coating, the concentration of an antibody, the concentration of the secondary antibody, the Na+ concentration, the pH value, and the methanol content. IC_50_ and F_max_/IC_50_ were used as evaluation criteria to select the optimal working concentration of the method.

### Specificity

Under the optimal working conditions, a series of gradient standard solutions of oxyfluorfen structural analogs were prepared, and the standard curves of oxyfluorfen and its structural analogs were established, respectively; IC_50_ values were calculated; and the cross reaction (CR) rates were calculated (CR%). The CR rate calculation formula is as follows:

$$\begin{array}{l} \text{CR}\left(\text{\%}\right)=\left[\text{I}{\text{C}}_{50}\left(\text{oxymethoxine}\right)/\text{I}{\text{C}}_{50}\left(\text{structural analog}\right)\right]\times 100 \end{array}$$

### Analysis of Spiked Samples

The added recovery rate and relative standard deviation (RSD) were used as TRFIA evaluation criteria to evaluate the accuracy and precision of the established TRFIA.

A 50, 100, and 500 ng/g oxyfluorfen standard was added to five matrix (the soil, apple, peach, pear, and grape). Three replicates for each concentration were designed, three parallels for each replicate were set, and a blank control was set.

### The Correlation of FPIA With HPLC

In order to verify the reliability of the established oxyfluorfen monoclonal antibody TRFIA to detect oxyfluorfen, samples after actual field application were collected, and the TBS solution with the optimal methanol content was diluted to a constant volume for TRFIA detection, and the GC method compared the accuracy and correlation of the results.

## Results and Discussion

### Preparation for Eu Markers

The statistical curves of fluorescence luminescence and protein ultraviolet absorption after purification of oxyfluorfen monoclonal antibody combined with Eu^3+^-DTTA are shown in Supplementary Figure S1A. The fluorescence curve in the figure clearly shows two peaks when the purified solution was collected in tube 17 and tube 21. The two peaks corresponded to the successfully labeled Eu-labeled antibody and the free Eu^3+^-DTTA that was not successfully labeled with the antibody. Due to the formation of chelates with antibody markers, the molecular weight of chelates was much greater than that of the unlabeled Eu^3+^ -DTTA, so the chelates were purified in Sepharose Cl-6B agarose gel column and eluted first. This conclusion could also be displayed by the ultraviolet absorption value of the protein in the blue line (Supplementary Figure S1A), and it could be seen that the protein peak was only a single peak, showing a trend of rising first and then falling, and the peak of the protein peak was the same as the first fluorescence. The peaks overlap, which proved that the successfully labeled Eu-labeled antibody was eluted first. The solutions were combined according to the number of collection tubes, and their labels were used for subsequent TRFIA method research. The statistical curves of fluorescence luminescence and protein ultraviolet absorption of goat anti-mouse IgG antibody combined with Eu^3+^-DTTA are shown in Supplementary Figure S1B. According to the values shown in Supplementary Figure S1B, 18–21 tubes of the purified solution were combined and used as a Eu^3+^-labeled secondary antibody for the follow-up TRFIA method study.

After the Eu^3+^-labeled antibody was successfully synthesized, the Eu-labeled secondary antibody was selected as a representative, and the synthesized labeled antibody was scanned by the SpectraMax M5 multifunctional microplate reader to determine the optimal excitation light spectrum and emission light spectrum. As shown in Supplementary Figure S1C, the detection mode of the TRFIA analysis method was finally determined: the excitation light is 340 nm, the emission light is 613 nm, and the detection delay time is 400 μs.

### Optimization of the TRFIA

**Figure 1:**
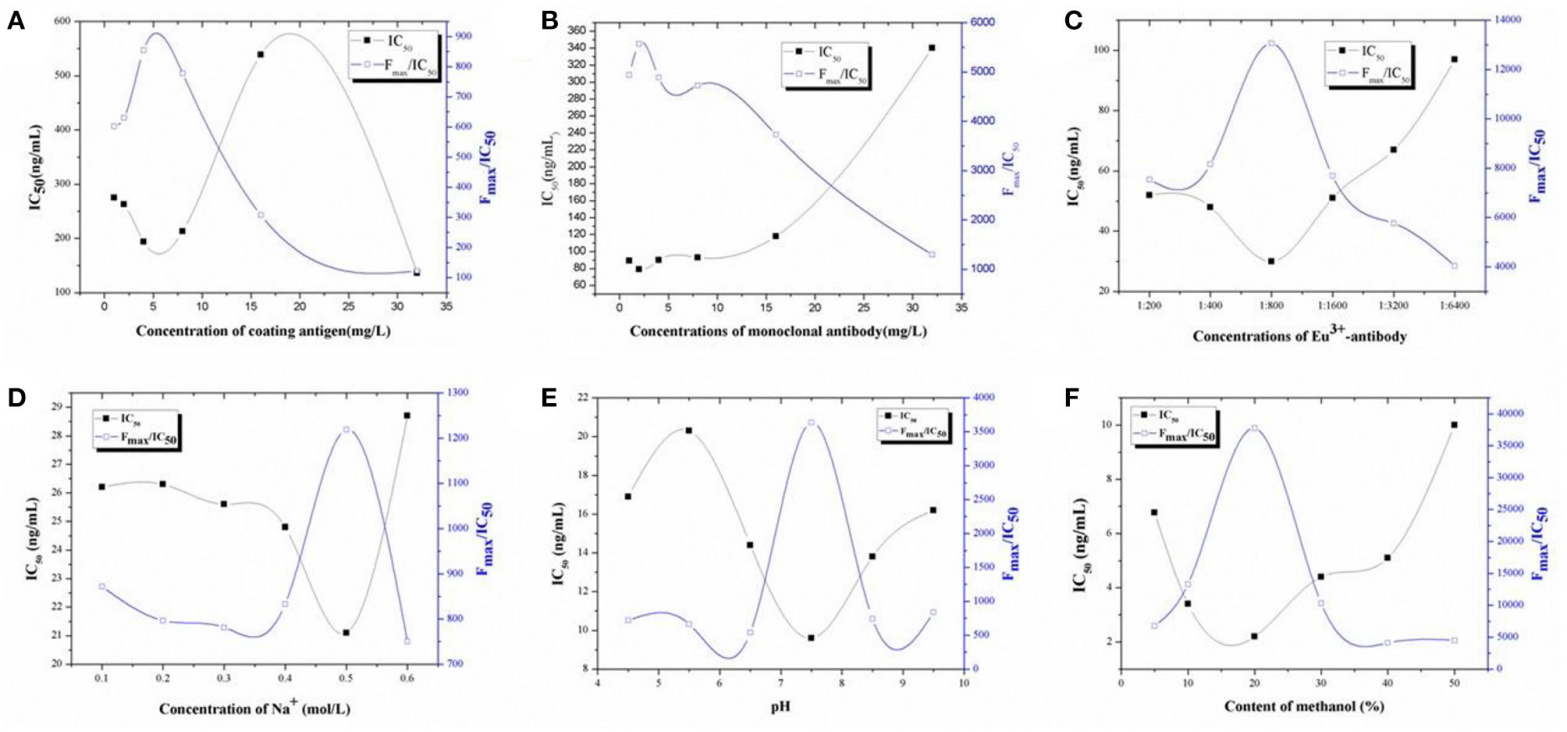
Optimization. **(A)** The optimization of the concentrations of coating antigen for TRFIA; **(B)** The optimization of the concentrations of first-antibody for TRFIA; **(C)** The optimization of the concentrations of Eu^3+^-antibody for TRFIA; **(D)** The optimization of the concentrations of Na^+^ for TRFIA; **(E)** The optimization of the pH values for TRFIA; **(F)** The optimization of the contents of methanol for TRFIA.

Because dc-TRFIA and ic-TRFIA use the same antibody and the same analyte, the optimal working conditions are basically the same, so all the working condition optimization data in this link are tested by the competitive TRFIA method. The concentration of the coating antigen, Eu^3+^-labeled GAR-IgG, and Eu^3+^-labeled McAb were optimized based on the higher F_max_/IC_50_ ratio and lower IC_50_. As shown in Figure 1, 4 μg/mL coating antigen (Figure 1A), 2 mg/L Eu^3+^-labeled McAb (Figure 1B), and the Eu^3+^-labeled GAR-IgG (Figure 1C) diluted 800 times were chosen for further research.

For solvent optimization, Na^+^ concentration, pH, and methanol could affect the sensitivity of the method. As shown in Figure 1D, the change of Na^+^ concentration had a great influence on the sensitivity of the method. The Na^+^ concentration was inversely proportional to the IC_50_ value, Na^+^ was increased, IC_50_ was decreased, and F_max_/IC_50_ was increased. When the Na^+^ concentration was 0.5 mol/L, the F_max_/IC_50_ was the highest, the IC_50_ value was the lowest, and the sensitivity was the highest. Therefore, the optimal concentration of Na^+^ was 0.5 mol/L. As can be seen from Figure 1E, when the pH was between 4.5 and 9.5, the method IC_50_ first rose and then fell and then rose; when the pH was 7.5, the IC_50_ of the method was the lowest, the sensitivity of the reaction was the highest, and the F_max_/IC_50_ value was the highest. Therefore, the pH of the buffer finally used in the subsequent experiments was 7.5. The effect of methanol on the sensitivity of the method is shown in Figure 1F. When the methanol content in the system was 20%, the sensitivity of the method was the highest, the F_max_/IC_50_ value was also the highest, the standard curve of the buffer configuration was the closest, and the influence of the organic solvent on the sensitivity of the reaction was the smallest. Therefore, the 20% methanol PBS solution was selected as the best working condition for subsequent experimental research.

Based on the optimization results of all working conditions, the final optimization result of TRFIA was obtained as the following test conditions: 4 μg/mL was selected for the coating, 2 μg/mL for the antibody, and 800 times for the second antibody. The working buffer was selected from TBS buffer containing 20% methanol, Na^+^ concentration of 0.5 mol/L, and pH 7.5.

### Sensitivity and Specificity

**Figure 2:**
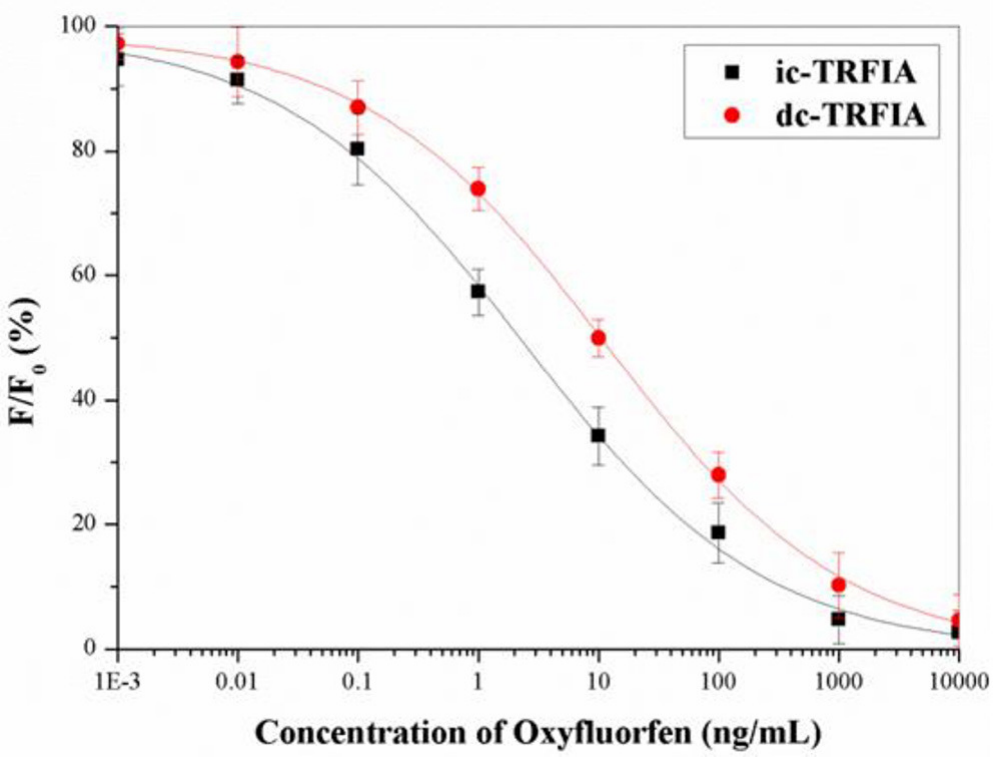
The standard curve of TRFIA for oxyfluorfen (The concentration of oxyfluorfen was 0.001, 0.01, 0.1, 1, 10, 100, 1,000, and 10,000 ng/mL).

Under the optimal conditions, the standard curve of oxyfluorfen TRFIA (Figure 2) was established. The IC_50_ of dc-TRFIA was 10.27 ng/mL, the LOD was 0.071 ng/mL, and the linear range IC_10_-IC_90_ was 0.071–1074.3 ng/mL; the IC_50_ of ic-TRFIA was 2.76 ng/mL, the LOD was 0.024 ng/mL, and the linear range IC_10_-IC_90_ was 0.024–504.6 ng/mL.

After comparing the two TRFIA analysis methods, it was found that the sensitivity of ic-TRFIA was nearly five times higher than that of dc-TRFIA, and the minimum detection limit is lower than dc-TRFIA. The sensitivity of ic-TRIFA and dc-TRFIA was different, which might be due to the different antibodies labeled with fluorescent markers. When the first antibody was labeled with a fluorescent label, it might affect the antigen-binding active site of the primary antibody, reduced its activity, and affected the binding reaction between the primary antibody and the antigen; labeling the secondary antibody had less impact. Therefore, ic-TRFIA was selected for further analysis.

Compared with the MRLs of oxyfluorfen are 0.02 mg/kg in Canada, and 0.01 in Food and Agriculture Organization (FAO) of the United Nations, the sensitivity of the TRFIA could meet the requirements for the detection of oxyfluorfen under an appropriate pretreatment. According to the published articles, the LOD values of ELISA, CELIA (Sheng et al., 2018), HPLC (Xiang et al., 2002), and GC (Calderon et al., 2015) were 4.8, 1.6, 7, and 50 ng/mL; the TRFIA were more sensitive than the above-mentioned methods.

### The Cross Reaction

The reaction rate of structurally similar compounds of oxyfluorfen was determined by ic-TRFIA (Table 1). The results showed that there was no significant cross-reactivity with other structural analogs (CR < 0.01%) except for some CR with benzofluorfen (CR = 11.58) and the bifenox (CR = 8.23%).

**Table 1:** The cross-reactivities of analogs related to oxyfluorfen by the ic-TRFIA.

**Compound**	**Structure**	**IC_**50**_ (ng/mL)**	**CR (%)**
Oxyfluorfen	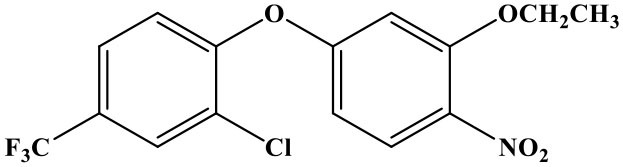	2.76	100
Benzofluorfen	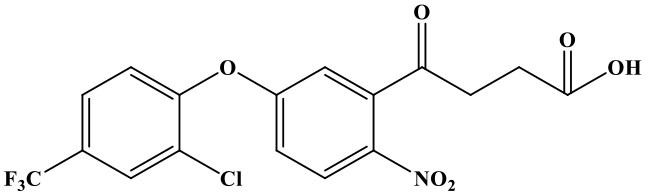	23.84	11.58
Bifenox	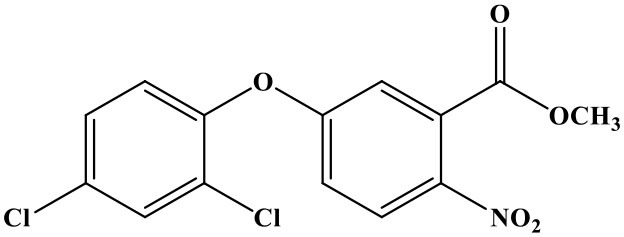	33.52	8.23
Fomesafen	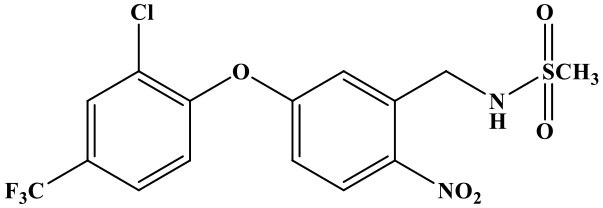	>30000	<0.01
Acifluorfen sodium	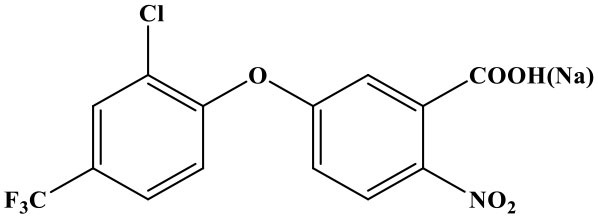	>30000	<0.01
Lactofen	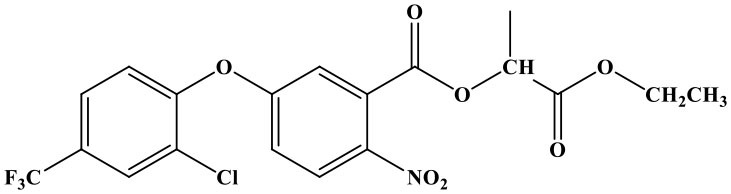	>30000	<0.01
Nitrofen	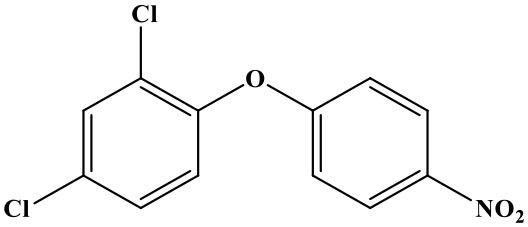	>30000	<0.01

### Matrix Effect and Recovery of Spiked Samples

The sample matrix could affect the accuracy of the immunoassay and was usually removed by dilution with buffer. As shown in Supplementary Figure S2, when the soil was diluted 20 times and the apple, pear, peach, and grape were diluted five times, the matrix effect no longer affects the detection sensitivity of the TRFIA method. The sample dilution factor obtained from the study was used for dilution of the later experimental sample. Under the dilutions, the average recovery of the established method in soil, apple, pear, grape, and peach was 74.6–108.3% (Table 2). The relative standard deviation is between 2.1 and 10.9%. The results were in line with the pesticide residue detection standards established in China. It was proved that the established ic-TRFIA method had extremely high accuracy and could quantitatively analyze the sample.

**Table 2:** Recoveries of samples spiked with Oxyfluorfen by TRFIA (*n* = 3).

**Sample**	**Spiked concentration (ng/g)**	**TRFIA**	
		**Mean recovery ± SD (%, *n* = 3)**	**RSD (%)**
Soil	50	96.5 ± 4.6	4.8
	100	97.4 ± 3.5	3.6
	500	74.6 ± 3.1	4.2
Grape	50	98.5 ± 2.1	2.1
	100	108.3 ± 4.9	4.5
	500	87.6 ± 7.4	8.5
Peach	50	100.6 ± 5.1	5.1
	100	107.8 ± 7.4	6.9
	500	103.1 ± 6.3	6.1
Apple	50	89.4 ± 6.9	7.7
	100	93.2 ± 6.6	7.1
	500	100.3 ± 6.8	6.8
Pear	50	76.9 ± 4.6	5.9
	100	86.4 ± 3.1	3.6
	500	79.7 ± 8.7	10.9

### The Validation of TRFIA With HPLC

**Figure 3:**
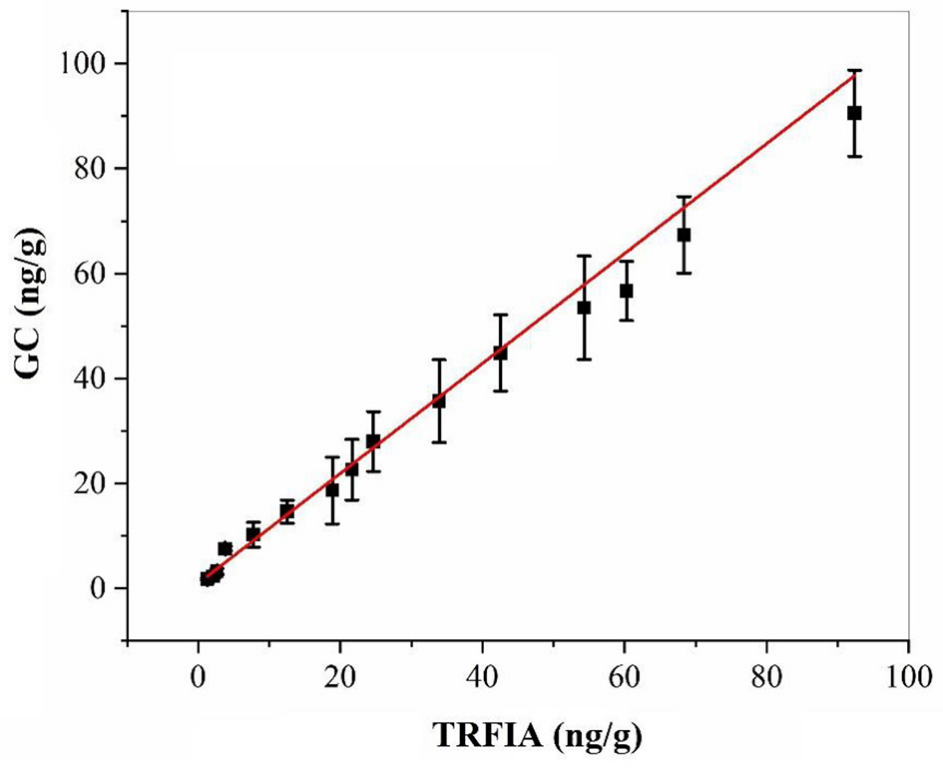
Correlation between TRFIA and GC for the concentrations of oxyfluorfen in authentic samples.

The residual amount of oxyfluorfen in the real sample of apple was detected by harvesting some real samples from the field, and the same sample was detected by GC at the same time. The correlation analysis between the two methods is shown in Figure 3, the correlation equations of the two methods was *y* = 0.975x – 0.4446, and the correlation coefficient *R*^2^ = 0.9901, which proved that the established oxyfluorfen monoclonal antibody TRFIA method had high accuracy and reliability.

## Conclusions

In this study, The TRFIA method established in this study used a time-resolved detection mode to minimize the interference of background fluorescence and improve the accuracy of the detection results. In the detection, a new fluorescent chelate compound with a higher fluorescence intensity can be formed with the labeled antibody, which greatly increases the fluorescence intensity, thereby improving the detection sensitivity of the method. Under the optimized conditions, the IC_50_ of the method was 2.76 ng/mL and the LOD was 0.024 ng/mL. The sensitivity of this method was 23.5 times higher than that of the previously established alloantibody ic-ELISA method, and the sensitivity of the CLEIA method was increased by eight times, which fully meets the requirements for ultra-sensitivity analysis of oxyfluorfen sample residues. Compared with instrumental analysis, the use of organic solvents was greatly reduced, and the operation is also simpler. The established method is applied to the analysis of the added sample and the actual sample, and the correlation verification with the GC method further ensures that the precision and accuracy of the method satisfy the analysis of the oxyfluorfen residue. The developed TRFIA provided an ultrasensitive, throughput, rapid, and economic method for large-scale screening and monitoring of oxyfluorfen residues in food environmental samples.

## Data Availability Statement

The original contributions presented in the study are included in the article/Supplementary Materials, further inquiries can be directed to the corresponding author.

## Author Contributions

ES: conceptualization, methodology, formal analysis, visualization, software, writing-original draft, resources, writing-original draft, writing-review and editing, supervision, project administration, and funding acquisition. YL and YT: validation, formal analysis, visualization, and software. YX and ZL: formal analysis. All authors contributed to the article and approved the submitted version.

## Conflict of Interest

The authors declare that the research was conducted in the absence of any commercial or financial relationships that could be construed as a potential conflict of interest.
