# Supplementary material for: Sensitive Time-Resolved Fluorescence Immunoassay for Quantitative Determination of Oxyfluorfen in Food and Environmental Samples
Source: Front Chem. 2021 Jan 7;8:621925. doi: 10.3389/fchem.2020.621925 (PMC7817953; doi:10.3389/fchem.2020.621925)
Supplement: Supplementary file 1 [file Data_Sheet_1.docx]

**Sensitive time-resolved fluorescence immunoassay for quantitative determination of oxyfluorfen in food and environmental samples**

En Ze Sheng^1a*^,Yu Ting Tan^2a^,Yu Xiao Lu^1^, Yue Xiao^1^, and Zhen Xi Li^1^

^1^ *Jiangsu Collaborative Innovation Center of Biomedical Functional Materials and Jiangsu Key Laboratory of Biofunctional Materials, School of Chemistry and Materials Science, Nanjing Normal University, Nanjing, 210023, P. R. China.*

^2^*Department of Pesticide Science, College of Plant Protection, Nanjing Agricultural University, Nanjing 20095,China*

*^a^: the two authors contribute equally to this article.*

^*^*Corresponding author. Tel.: +86 25 85891051. E-mail address: shengenze@sina.com*.

**Detection of oxyfluorfen.** For GC, a 20 g sample, 10 mL water and 60 mL acetonitrile were mixed for 1 h. The organic phase was concentrated, then diluted with 2 mL of acetone and analyzed by GC-ECD. One DB-1 (30 m × 0.32 mm × 0.25 µm) column was used. The column temperature was initially held at 150 °C for 2 min and then raised to 210 °C by 6 °C/min. Finally, the temperature was raised to 270 °C by 30 °C/min, and maintained this value for 6 min. The carrier gas was nitrogen (58 mL/min) and the detector was an ECD at 320 °C.

**Analysis of spiked samples.** All food samples were obtained from Walmart. All environmental samples (soil) were obtained from the farm in Nanjing Agricultural University. All samples were verified as not containing oxyfluorfen before the spiking and recovery studies by GC were performed.

**Real samples detection.**The sample matrix could affect the accuracy of the immunoassay and was usually removed by dilution with buffer and the extracted steps were as the same as real samples detection. The proposed fluorescence method was used for oxyfluorfen detection in food and environmental samples. The samples (20 g) were mixed with 50 mL acetonitrile, ultrasonic extraction for 10 min and centrifuged for 10 min at 4000 rpm. Afterwards, the contents were filtered through anhydrous sodium. Afterwards, the contents were filtered through anhydrous sodium sulfate. The extraction procedure was repeated for three times, and the extract was combined. The organic phase wasevaporated to dryness. The remainder was dissolved with 2.0 mL Tris buffer solution containing 10% ethanol, and the oxyfluorfen concentration in real sample was detected by using the current method based on calibration curve.

**Figure captions:**

**Figure S1.** Preparation for Eu markers

**Figure S2.**The matrix effect of samples on the sensitivity of the oxyfluorfen.


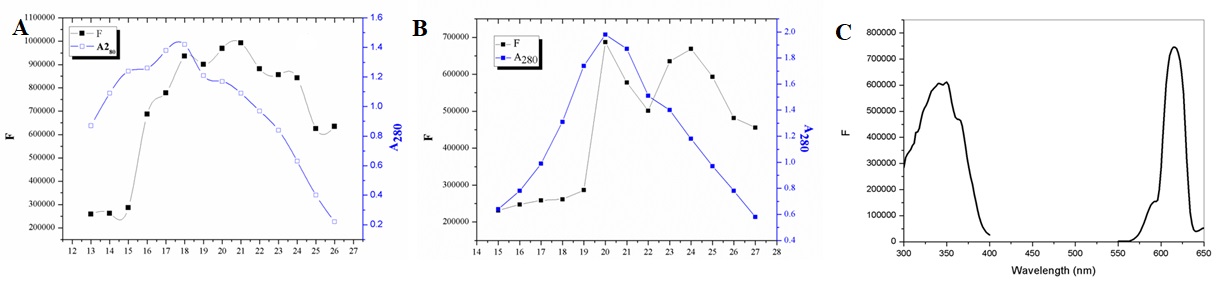


**Figure S1.** Preparation for Eu markers. A: The separation curves of Eu^3+^-DTTA with oxyfluorfen McAb; B: The separation curves of Eu^3+^-DTTA with IgG; C: The fluorescence spectrum of Eu^3+^-antibody.


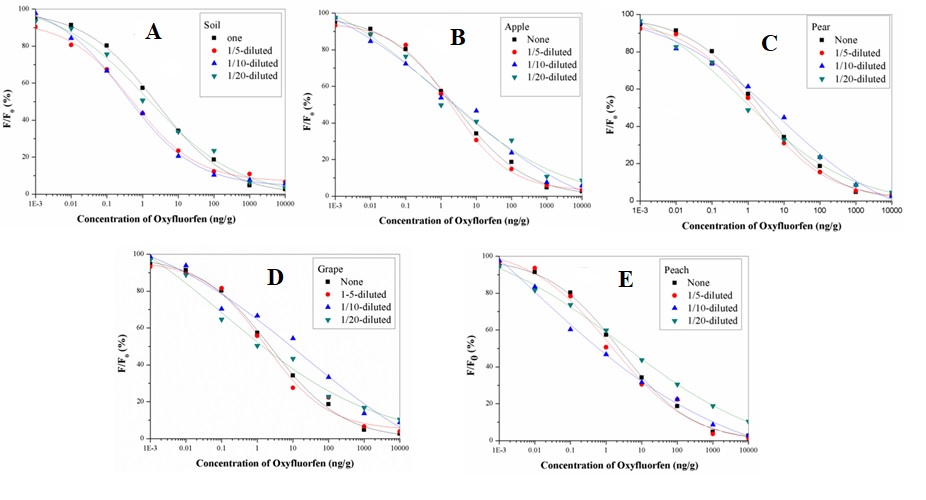


**Figure S2.**The matrix effect of samples on the sensitivity of the oxyfluorfen.
